# French Experience with Buprenorphine : Do Physicians Follow the Guidelines?

**DOI:** 10.1371/journal.pone.0137708

**Published:** 2015-10-19

**Authors:** Morgane Guillou Landreat, Charles Rozaire, Jean yves Guillet, Caroline Victorri Vigneau, Jean Yves Le Reste, Marie Grall Bronnec

**Affiliations:** 1 Department of addictive disorders, ERCR SPURBO,, Université de Bretagne occidentale, Brest, France; 2 Addiction psychiatry, CHU Nantes, Nantes, France; 3 Addiction department “Les Apsyades”, Nantes, France; 4 Pharmacology department, Universitary Hospital Nantes, Nantes, France; 5 EA 4275, Faculté de médecine Nantes, Nantes, France; 6 Department of general practice, ERCR SPURBO, Universitéde Bretagne occidentale, Brest, France; 7 Addiction psychiatry, Universitary Hospital Nantes, Nantes, France; Peking University, CHINA

## Abstract

Opiate dependence affects about 15,479,000 people worldwide. The effectiveness of opiate substitution treatments (OST) has been widely demonstrated. Buprenorphine plays a particular role in opiate dependence care provision in France. It is widely prescribed by physicians and national opiate substitution treatment guidelines have been available since 2004. In order to study the prescribing of buprenorphine, we used a questionnaire sent by email, to a large sample of physicians. These physicians were either in practice, or belonged to an addiction treatment network or a hospital. The main objective of this work was to measure the extent to which the theoretical, clinical attitude of physicians towards prescribing buprenorphine (BHD) complied with the statutory guidelines. We showed that the physicians we interviewed rarely took into account the guidelines regarding buprenorphine prescription. The actual prescribing of Buprenorphine differed from the guidelines. Only 42% of independent Family Physicians (FPs), working outside the national health care system, had prescribed buprenorphine as a first-time prescription and 40% of FPs do not follow up patients on buprenorphine. In terms of compliance with the guidelines, 55% of FPs gave theoretical answers that only partially complied with the guidelines. The variations in compliance with the guidelines was noted according to different variables and took into particular account whether the physician were affiliated to a network or in training.

## Introduction

Opiate dependence, whether it involves legal or illegal drugs, is a major public health issue worldwide. Opiate dependence affects about 15,479,000 people worldwide [1]. In Europe, the average prevalence of problem opiate users is estimated at between 3.6 and 4.4 per thousand inhabitants (15–64 years old). The effectiveness of Opiate substitution treatments (OST) such as methadone, buprenorphine or buprenorphine/naloxone has been widely demonstrated [2–4]). In France, buprenorphine plays a particular role in opiate dependence care provision. This role is rooted in history and in the consensus against opiate substitution that lasted well into the mid-90s in France.

Treatments for other chronic pathologies normally obtain Marketing Authorization (MA) after scientific appraisal, in which, inter alia, the risk/benefit relationship is assessed. Buprenorphine, however, obtained MA in an emergency situation where there was political and ideological upheaval, mainly linked to the HIV epidemic [5,6]. A unique system was then implemented: more than 30 years after Dole’s and Nyswander’s work [7], non-experimental methadone centers were set up, with limited access for patients. Since 1995, methadone has been prescribed only by specialized physicians in addictive disorders centers or in hospitals (since 2001). FPs can only prescribe methadone after referral from a specialized center. The key issue in this system was the implementation of buprenorphine prescription [2,4,6].

A unique feature of the French treatment system is that all registered physicians are allowed to prescribe buprenorphine without any special education or licensing. Once prescribed, buprenorphine can be delivered by any pharmacy chosen by the patient and named on the prescription.

This system rapidly proved to be efficient in terms of public health, access to care and risk reduction [2,6]. 170,000 patients are currently being treated for opiate dependence, 65% of whom are on BHD, and 35% on methadone [8]. Nevertheless the fact that buprenorphine is so accessible has facilitated misuse. Observing the types of misuse, and the associated damage, led the authorities to issue recommendations for good practice in substitution treatments. In 2004, the Heath Authority (HA) brought together experts on opiate substitution in order to issue national guidelines on therapeutic strategies for opiate dependent subjects [9].

These guidelines focus on stabilizing and improving the patients’ quality of life [9] They still apply today and are still validated by national experts. However, almost 10 years after these recommendations were issued, a large number of physicians are still reluctant to prescribe OSTs, or only consider prescribing them as a transitional measure before abstinence [10]. According to the 2009 Health Barometer in Family Medicine, 35.7% of interviewed FPs did not accept opiate dependent patients [11]. This reluctance is often explained by the fear of misuse or, to a greater extent, by the fear of abetting a type of behavior judged as morally reprehensible. Indeed, although in 85% of cases, FPs estimated that opiate dependence is primarily a disease, 90% of them are distrustful and think that opiate dependent patients are not compliant [12].

This diversity in interpretation and application of the guidelines generates inconsistent care provision that is detrimental to patients, especially in terms of OST access and follow-up.

We have reflected upon this diversity in the prescribing of buprenorphine within a legal context which still allows buprenorphine to be as widely available for prescription as any other treatment.

The study was conducted with physicians, whose characteristics varied in terms of education, location and type of practice, OST prescription practices, theoretical compliance with the statutory national guidelines (9), and their attitudes towards OST.

## Material and Methods

### Main objective

The main objective of this work was to measure the extent to which the theoretical, clinical attitude of physicians towards prescribing buprenorphine (BHD) complied with the statutory guidelines [9].

### Secondary objective

The secondary objective was to identify factors that favor or limit the extent to which the clinical attitudes comply with the recommendations.

### Ethics statement

The French legislation on biomedical research does not require the competent authorities’ authorization or ethics committee approval for this type of research. However, Nantes UH’s General Management Department and the Faculty of Nantes validated this clinical research.

### Population

#### Inclusion criteria

- Independent FPs, belonging to an addiction network or otherwise, or employed at an addiction clinic
- Available IT equipment
- Internet access
- Willing to participate in the study

#### Exclusion criteria

- Non prescribing physicians
- No IT equipment available
- No Internet access
- Not willing to participate in the study

Three samples of physicians were selected with the intention of representing the 3 prescribing groups within the whole range of BHD prescribers. It was not possible to belong simultaneously to 2 samples.

- **Group 1:** a group of in-practice FPs whose practices did not focus solely on addiction (« FP » group). This group was the control group, which served as a reference for measuring the effect that belonging to the other, more addiction-focused groups had on the quality of BHD prescription practices. This group was formed using contact information provided by the Family Practice Department at Nantes faculty of medicine. They had a mailing list comprising all the in-practice FPs who had agreed to participate in studies. Those belonging to an addiction network or those treating addiction in a hospital setting were excluded.
- **Group 2:** a group of in-practice FPs, belonging to an addiction network (« Network » group). It was formed from two networks, namely the RTRN (Loire Atlantique) and the ICARES (Charente Maritime) networks. These networks were recognized by the French Health Ministry. Only physicians specially trained to deal with addiction belong to these networks. Those treating addiction in a hospital setting were excluded.
- **Group 3:** a group of FPs, psychiatrists or gastroenterologists practicing in a hospital setting, treating patients with an addictive pathology (« Hospital » group). They provided ambulatory care in opiate maintenance. This group was formed using the 2008 Alcohol Treatment care structures Directory (6^th^ edition) which comprehensively inventories addictive disorders care establishments treating addiction (2 Universitary hospital, 7 general hospital and 7 ambulatory addictive disorders care centers). The target consisted of all specialized care establishments in the Pays de la Loire region.

#### Questionnaire

The Family Medicine department, the addiction networks and the Addiction Psychiatry department, working as a team, constructed the questionnaire.

This questionnaire was divided into three parts:

1. The first part determined the prescriber’s profile through socio-demographic data (age, sex,…), professional data (specialty, type of practice: independent–/hospital/network), BHD prescription habits data (having prescribed BHD as a first-time prescription, number of patients followed-up on BHD) and data on the perceived relationship with opioid dependent patients, and the perceived quality of information received on OSTs.
2. The second part of the questionnaire determined the extent to which the theoretical, clinical attitudes towards a clinical case complied with the guidelines [HA, 2004].
  A steering committee was set up, composed of several addiction therapy professionals who were trained in OSTs. They built a case report, by consensus, based on recommendations.
  First 7 main focal points were extracted from the HA guidelines (2004).
  - Recommendation #1: The patient must be the one to initiate the discontinuation of the treatment (« The request to stop OST can only come from the patient himself »)
  - Recommendation #2: There is no optimal duration for a substitution treatment (« There is no optimal duration for an OST »)
  - Recommendation #3: The OST can be progressively reduced (« Experience shows it is possible to discontinue treatment, slowly and gradually. »)
  - Recommendation #4: Discontinuation terms must be arranged with, and agreed by, the patient (« The terms of the decrease must be managed by the patient himself »)
  - Recommendation #5: The treatment requires constant adjustment (« It is unrealistic to fix the duration in advance of the decrease process »)
  - Recommendation #6: Specific vigilance must be implemented during the discontinuation process (« No reliable criterion can allow predictions of success or failure to be made when attempting to discontinue an OST »)
  - Recommendation #7: It is important to find situations which favor discontinuation (« Some situations that are more favorable than others: social inclusion, long-term discontinuation of non-prescription substances, etc. »)
  We have not kept recommendation #3: since no decreasing protocol is consensual. This question remains heavily debated in the literature. « Guidelines» are therefore not binding in this particular area.
  Recommendation #7 advocates a « favorable situation for discontinuation ». This focus on context implies that dependence should be submitted to a global medico-psycho-social evaluation. Given that this variable seemed essential in therapeutic decision-making, an element regarding addictive pathology evaluation was added.
  These 7 variables were aggregated in a case report, presented as a multiple-choice form to be filled out on the Internet. It was not possible to go back to previous questions. For each question, there were 3 proposed answers: one that complied with the guidelines, and two that did not. The two non-compliant answers, for quotation purposes, were distinguished as non-compliant but debatable answers and those non-compliant answers which actually contradicted the guidelines.
3. The third part determined the physician’s attitude toward OST. It explored the physician’s feelings in two areas. The first explored whether OSTs were considered to be a treatment or a drug. The second explored whether OSTs were considered an individualized treatment or a public health instrument.

#### Questionnaire administration method

An explanatory email about our study was sent to the email addresses we had previously gathered from the FP group, the network group (N) and the hospital group (H). The email gave details about the context and objectives of the study, along with a link that led to the dedicated web based platform, where the questionnaire was embedded. Filling out the questionnaire took 5 to 10 minutes.

### Statistical analysis

#### Questionnaire structure

Compliance scores were calculated using cumulated compliant answers, weighted to take into account non-compliant answers (simple, questionable, or contrary to recommendations).

- Compliant answers: +1
- Non-compliant answers: questionable = 0, simple = -2,
- Contrary to recommendations = -5

Theoretically, scores were spread from -23 to +7. Predefined categories for compliant practices were set out. From -23 to 0 = group whose practices partially complied with recommendations.

From 1 to 3 = group whose practices complied with recommendations. Possible scores were 3 (5 « +1 » answers, 1 « 0 » answer, 1 « -2 » answer), 2 (4 « +1 » answers, 2 « 0 » answer, 1 « -2 » answer) and 1 (6 « +1 » answers and 1 « -5 » answer, or 5 « +1 » answers and 2 « -2 » answer).

From 4 to 7 = group whose practices complied, in most respects, with the recommendations. This group did not, therefore, include non-congruent answers that were contrary to the spirit of the text (-5). Possible scores were 7 (7 « +1 » answers), 6 (6 « +1 » answers, 1 « 0 » answer), 5 (5 « +1 » answers and 2 « 0 » answers) and 4 (6 « +1 » answers and 1 « -2 » answer).

A quantitative and qualitative descriptive analysis was then conducted, using averages and standard deviations. The compliance with prescription guidelines scores were calculated, including the number of answers which exactly matched the guidelines, which indicated global performance.

Scores were analyzed, both descriptively with means, and quantitatively with scores and categories. They were also weighted to take into account negative answers.

A comparative analysis of global performance scores was conducted, to make sure that the observed differences were not linked to the weighting.

The influence of the various factors (socio-demographic data, continuous training and volume of OST treatment undertaken) on compliance with the substitution prescription guidelines was then investigated, using Chi^2^ to test the independence of the global sample.

## Results

### Descriptive analysis

542 physicians were contacted via email: 356 belonged to the FP group, 78 belonged to the Network group and 108 belonged to the Hospital group. The response rate was of 44%, with 193 questionnaires collected: 111 from the FP group (response rate 31.1%), 37 from the Network group (response rate 47%) and 45 from the Hospital group (response rate 41.6%). Socio-demographic and professional characteristics are reported in Table 1 below.

**Table 1:** Socio-demographic and professional characteristics of responding physicians.

	FPs	Network	Hospital
Respondents (N)	111	37	45
Mean age	49,4	51,7	46,2
Specialty			
FP	100%	100%	47%
Psychiatrist	0	0	40%
Gastroenterolologists	0	0	13%
M/W Ratio	2.23	3.17	0.45
Addictology formation	38%	81%	87%
Number of patients on BHD			
6 and more	5%	65%	45%
From 2 to 5	34%	16%	19%
1	15%	16%	10%
0	45%	3%	26%
First-time BHD prescription° practice in general	42%	95%	78%
First-time BHD introducers among prescribers°°	67%	97%	85%

°**First-time BHD** prescribers are physicians who introduced a BHD treatment at least once.

°°Prescribers are physicians with at least one patient currently treated on BHD

In the three groups, the percentage of physicians who had at least one patient being prescribed buprenorphine was as follows: 55% FPs (N = 60), 97% Network (N = 36), 74% Hospital (N = 33).

Among the three groups of physicians who had at least one patient on BHD, the percentage of physicians who were treating 6 or more patients with BHD was as follows: 9% FPs (N = 5), 67% (N = 24) Network, 61% (N = 20) Hospital.

#### Ease of contact with opioid dependent patients

The results regarding how easy the follow-up of opioid dependent patients was felt to be are reported in Table 2.

**Table 2:** Ease in opioid dependent patients follow-up.

	FPs	Network	Hospital
Moderately or completely at ease	37%	92%	82%
Moderately or not at all at ease	50%	8%	15%
Not applicable	13%	0%	5%

65% of FPs considered medical information received regarding OSTs as inadequate for their practice needs. 85% of hospital physicians considered it adequate, as did 98% of network physicians.

#### Analysis of the compliance with recommendations

**Fig 1:**
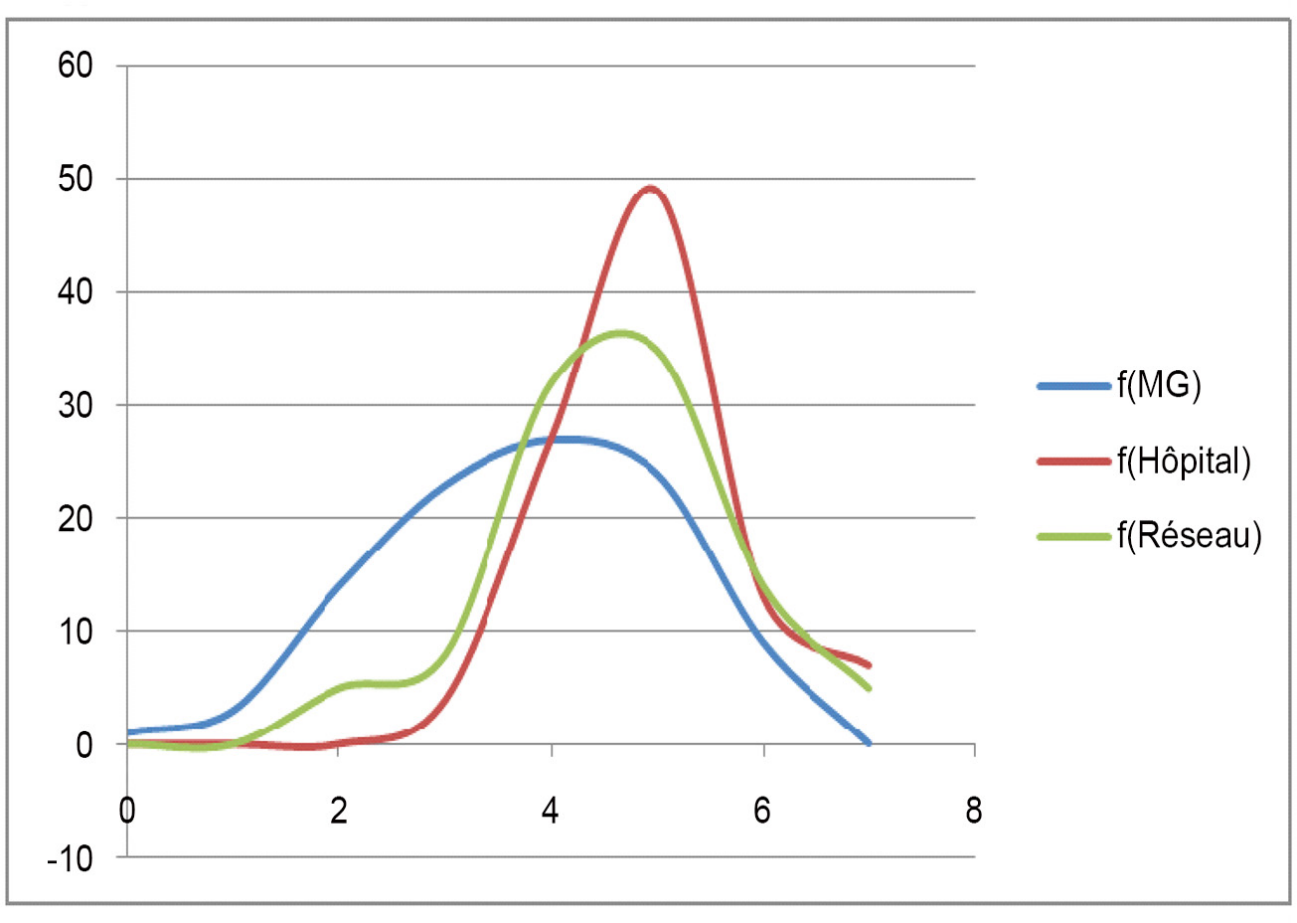
Distribution of the congruent answers between professional categories(MG = Fps, Hopital = Hospital, Réseau = Network).

Fig 1 shows the distribution of average global scores for compliance (not weighted to take into account non-compliant answers) according to the 3 groups: FPs group, Network group and Hospital group. This distribution is congruent and the average distribution differs among the three groups.

The 3 samples present a normal distribution curve, with an average close to 5 exact-match answers for the « Network » and « Hospital » samples and an average of 4 for the « FPs » sample, and unequal variances between the samples: Var (FPs) > Var (Network) > Var (Hospital).

Table 3 presents the distribution within the compliance categories: partially compliant (PC), Mainly Compliant (MC), Compliant (C) according to the compliance scores weighted to take into account the non-compliant answers.

**Table 3:** Distribution of the FPs, Network and Hospital groups within weighted scores compliance categories.

Category (scores)	PC (-23–0)	MC (1–3)	C (4–7)
FPs	55%	18%	27%
Network	14%	35%	51%
Hospital	20%	36%	44%

The information received was perceived as « moderately » or « completely » sufficient by respectively 85% and 98% of the « Hospital » and « Network » groups, vs 36% for the « FPs » group.

The information received was perceived as « moderately » or « completely » appropriate by respectively 83% and 92% for the « Hospital » and « Network » groups, vs 40% for the « FP » group.

### Comparative analysis

The comparison of the unweighted compliance averages within the control group FPs is presented in Table 4. Significant differences between the FPs’ averages and the Network and Hospital averages are observed. There were no significant differences between the averages of the Network and Hospital groups.

**Table 4:** Comparison of average compliance scores according to professional categories.

	FPs	Network	Hospital
Means	3.81	4.59	4.91
Variances	1.74	1.36	0.86
Comparison with FP mean (p unilateral)	—	< 0.01	< 0.01°
Comparison Network average (p bilateral)	—	—	0.17°°

° Given the statistical inequality of variances (Snédécor F test), A comparison of averages test for unequal variances (Welch test) was undertaken.

°° The test used was a bilateral test as the direction of the difference was unknown in advance.

Regarding the impact of variables on the extent to which therapeutic attitudes matched recommendations, belonging to a compliant group was independent of socio-demographic variables. Continuous training in self-reported addiction therapy had no impact on the compliant group (Table 5).

**Table 5:** Compliance in three groups of prescribers, according to number of patients followed up with BHD treatment.

	Numbers of patients With BHD (n)	PC	MC	C
FP	n>2	39%	27%	34%
	n<2	66%	12%	22%
Network	n >2	17%	37%	47%
	n<2	0%	29%	71%
Hospital	n>2	19%	37%	44%
	n<2	20%	33%	47%

Regarding the Network and Hospital groups, the number of patients followed up by physicians does not seem to point towards an effect size. Regarding the FPs group, prescribing physicians who had prescribed BHD to more than 2 patients, were more efficient (61% complied closely or very closely with the guidelines) than non-prescribing physicians or physicians who prescribed BHD to fewer than 2 patients (34%).

## Discussion

The main result of this study shows that physicians we interviewed rarely took into account guidelines regarding buprenorphine. These results were in line with what can be found in literature regarding the difficulties encountered by physicians in following guidelines on BHD or BHD/Naloxone[[4,11]. We are unable to know for certain whether this was due to difficulties in applying the guidelines or ignorance of the guidelines or rejection of these guidelines by the physicians. However, most countries (USA and Australia, among others) only recently introduced BHD or BHD/Naloxone, whereas in France this treatment has been available since 1996, and guidelines date back 10 years.

One of the main points of guidelines is to ease access to treatment with buprenorphine. Despite the fact that the regulatory prescription framework is extended to include any physician, it is noticeable that access to buprenorphine is not systematic. The population of physicians we looked at was representative in terms of the socio-demographic characteristics of the national population of FPs. Only 42% of independent FPs prescribed buprenorphine as a first-time prescription, and 40% of FPs do not follow up patients on buprenorphine.

These results were also in line with other studies [13,14]. However, their results differ from those of a previous study conducted in Family Medicine in 2009, where two thirds of the FPs interviewed declared that at least one opioid dependent patient had come in for a consultation over the previous year. These differences could be explained by methodological differences (FPs were interviewed over the year, and mostly by telephone) and age criteria (the population interviewed was older) but mainly it seemed possible to assume that some FPs followed up patients on BHD without getting involved in the prescription because they considered this treatment as different from others. Furthermore, as put forward in the literature, many physicians considered that the objective of managing opioid dependence is complete abstinence, via withdrawal, without OSTs [10], in spite of a bad prognosis on long term opiate withdrawal [14].

In terms of compliance with guidelines, 55% of FPs gave theoretical answers that only partially complied with guidelines. The differences in compliance with guidelines were noted according to different variables, and particularly according to affiliation to a network. The Network group has a 51% rate of answers which comply with recommendations, which is significant compared with the FPs or the Hospital group. The second variable that influenced the level of compliance to guidelines is the number of patients on BHD followed up by the physician. There was a clear effect after 6 patients, but that effect was already apparent after 2 patients. The more frequently physicians prescribe buprenorphine, the more comfortable they are prescribing it again which, in turn, increases their pharmacological and technical prescription skills. It is also possible to highlight that the physicians’ perceptions of their opioid dependent patients changed, based on the number of patients and on affiliation to a network [15]. In this study we address addictions, but other studies have shown that belonging to a care network for HIV, hepatitis or addictions, fosters the development of substitution practices and strengthen these practices [13].

Risk reduction and the definition of personalized care projects are among the fundamental values shared in these networks. Differences were found in perceived therapeutic objectives between the Network, Hospital and FPs groups. The physicians most involved in opioid dependent patients’ care were Network and Hospital physicians, and they clearly considered BHD a tool for personalized treatment, whereas FPs considered it as a social control tool [15]. Conversely, there was no difference in perception between the perception of BHD as a drug or the perception of BHD as a treatment. As was found amongst patients, the prescribers are ambivalent towards BHD, whether it be perceived as a drug or as a treatment [15,16].

On average, physicians declared a high rate of participation in continuous addiction therapy training. 38% of FPs and more than 80% of Network or Hospital physicians reported having attended continuous medical training (CMT) in addiction therapy. This prevalence is in contrast to previous studies which reported 33% CMT among BHD prescribing FPs [13]. On one hand, the declarations could be overestimated to enhance social desirability, on the other hand, some physicians may have attended training in an addiction field other than that specific to opiate dependence. But contrary to our assumptions, there was no statistically significant effect of self-reported CMTs on practices which complied with guidelines.

Regarding accessible and available medical information on opiate substitute treatments, FPs and Hospital and Network physicians have very different views on care. More than 60% of independent FPs considered that the type of medical information available on OSTs was insufficient and inadequate to their needs. Conversely, Network and Hospital physicians were mainly satisfied with available information Existing information materials seemed inadequate for Family Medicine, or were not displayed, handed out or accessible enough. This lack of access to information was identified as a limiting factor to OST access for patients, according to FPs [17]

This study had some limitations, which may have had an impact on the interpretation of the findings. First, the national guidelines were written 10 years ago and could lack clarity, leading to a possible information bias. Second, there are several debatable points in the guidelines, which limits their application in real-life situations, leading to a confusion bias. But our statistical analyses addressed this point and found that experienced physicians’ practices matched national guidelines. Third, the theoretical, clinical attitude which was expected did not take into account the adjustments that are sometimes necessary in reality, leading to another confusion bias.

Organizing patients’ care under OST was thus stratified. In this respect, it was possible to distinguish between the following types of physicians: non-prescribing physicians, who may receive patients on BHD or MTD in consultations but who did not get involved in their treatments; physicians who rarely prescribed BHD or MTD, whose practices only moderately complied with the recommendations and physicians who often prescribed BHD or MTD, who may or may not belong to a network or hospital. This stratification seemed organized, but certainly lacked transparency for patients.

This study highlights the limitations of the French system and the wide availability of buprenorphine treatments, which matches the findings of other recent studies [17]. Almost 20 years after marketing authorization, France is far from being in a situation where all physicians prescribe buprenorphine, with 40% of physicians not prescribing it at all. It is impossible to deny the benefits of such a system, in terms of public health, as has been highlighted in previous work, concerning mortality and morbidity rates [2,15] But the limitations are also substantial. A minority of physicians is responsible for the majority of the buprenorphine prescribed. FPs play a central part in treatment accessibility, but their profile seems specific: they are interested in patient-centered care which is a core competency of Family medicine according to Wonca [18]. That competency has recently been assessed in most French universities and results could be even higher in the next decades. We have to consider that physicians involvement in networks, and buprenorphine prescription experience, influence their ease and their compliance with the recommendations. They followed up several patients on BHD, given that ease and compliance with recommendations that are proportional to the physician’s experience and to his/her involvement in networks. Risks linked to non-compliance with prescription recommendations are many: under-dosing, ineffective treatment [19], « switching addiction », and misuse.

Buprenorphine misuse, by snorting or injecting, involved 10% to 30% of patients treated with buprenorphine [17,19,20,21] According to recent studies, 12.5% to 16.9% of buprenorphine reimbursement in France was destined for the black market [21]. 82% of physicians feel concerned about misuse and the diversion of medicines, according to the Benyamina study [17]. But the introduction of buprenorphine/naloxone in January 2012 may help reduce the diversion of buprenorphine [22]

Since undertaking this work, we find it deplorable that addictive disorders networks, previously under threat, have now been disbanded in France. Despite access to buprenorphine in primary care, the management of opioid dependence may well remain the concern of specialists in the field, and the development of studies into opiate dependence in Family Practice, involving primary care stakeholders, will help to re-establish opiate dependence care within Family Practice.

## Conclusions

With the objective of improving opioid dependent patients’ care, and improving BHD use, it might be pertinent to formalize a continuum of care for patients on BHD. This could be achieved through BHD accreditation, as contemplated for methadone on the streets, or at least a treatment program, as is occasionally recommended for new treatment management of alcohol dependence. Strengthening the role of networks could be an alternative path, as could the selection of FPs trained in the competency of patient-centered care. Nevertheless the actual results, in mortality and morbidity rates, should be kept in mind to avoid “throwing the baby out with the bathwater” by introducing an inadequate reform.

## Supporting Information

S1 Text Questionnaire anglais .doc 'english version of the questionnaire'. (DOC)

S2 Text 'Letter from the universitary Hospital of Nantes -ethics statement'. (DOC)

S3 Text Questionnaire VO French.doc 'French version of the questionnaire’. (DOC)
