# Supplementary material for: French Experience with Buprenorphine : Do Physicians Follow the Guidelines?
Source: PLoS One. 2015 Oct 19;10(10):e0137708. doi: 10.1371/journal.pone.0137708 (PMC4610705; doi:10.1371/journal.pone.0137708)
Supplement: S1 Text — (DOC) [file pone.0137708.s001.doc]

The physicians received an e-mail where the study was shortly explained, there was a link to the study platform. The link led to this page :

**Dear doctor,**

**We lead a study on buprenorphine prescription in Family pratice.**

**Many studies underline buprenorphine prescription interest in morbidity and mortality reduction in opiate dependent patients. But data are not clear, concerning indications, treatment duration or how to stop treatment. It may cause troubles for physicians face to buprenorphine prescription.**

**Our main objectives are :**

- **evaluation of theoretical buprenorphine prescription in a case study**
- **evaluation of buprenorhine perception by physicians**
- **definition of improvement axis in opiate dependant patients care in family practice**

**This study was led with the universitary addictology center of Nantes and the addictology network of Nantes , with the help of the universitary department of family practice of Nantes.**

**If you accept to answer, it will take you 5 minutes, all the data are totally anonymous.**

**When the study will be over, all the physicians who answer the questions will be informed by e-mail of the study results.**

**Many thanks for your participation.**

After this text, the physicians who decide to, had to go ta an item **« Answer the questionnaire »** and then the questionnaire started.

**How old are you ?**

**35-45 45-55 - 35 +55**

**Are you :**

**man woman**

**What is your practice ?**

**Family practice Psychiatrist Other : wich one :**

**Do you have a specific addictology formation ? (universitary )**

**yes no**

**have you already initiated a buprenorphine treatment on your own ?**

**Yes no**

**How many opiate dependent patient treated by buprenorphine do you regularly see for their treatment ?**

**1 2-5 +6 none**

**Do you feel at-ease in opiate dependent care ?**

**Yes, absolutely rather yes rather no not at all non concerned**

**Do you think that you have access to enough informations concerning opiate dependent patients treatment ?**

**Yes, absolutely rather yes Rather no not at all**

**Do you think that these informations match with your needs and your practice ?**

**Yes, absolutely rather yes Rather no not at all**

**About a case study**

**1. A young man (27 years old) (opiate dependent since 3 years) comes to see you. He is recently treated by buprenorphine (subutex ®) 12 mg/day . He is asking you about the duration of his treatment ? What do you answer ?**

- **a few months**
- **around 3 years**
- **other answer**

1. **He comes to see you regularly. You wish to measure his evolution. According to you which informations seems important to really measure addictive disorders evolution :**

**- Psychoactive drugs consumption or stop**

**- mutifactorial evaluation including social, familial, status and affective life**

**- patient's care compliance**

1. **Addictive disorders improvment is long. Among these variables, which one can we trust to measure the ability to stop substitution treatment , according to you ?**

**- No opiate craving**

**- No opiate consumption (except prescribed treatment)**

**- A satisfying social and professionnal status**

1. **This patient has moved. He comes back to see you 5 years later. He is well, he works, he has a good quality of life. He has still buprenorhine 4 mg per day, he is compliant. He asks you to renew his prescription. What do you say to him ?**

**- I think you have to continue this treatment, it seems it helped you to get better.**

**- Have already think about buprenorphine stop or regression ?**

**- Actually, I do not see buprenorhine indications anymore, I think we can start thinking about a buprenorphine stop**

- - 1. **You decided with the patient to try to stop buprenorphine. What is your behavior ?**

**-Discuss and decide with the patient of different ways to decrease ant to stop the treatment**

**-Quickly stop the treatment. Daily dose is weak.**

**-You define a strict protocol with slow tapering , with an abstinence objective predefined.**

- - 1. **You led a decrease , he has now 2.4mg/day. The patient comes to see you, he seems to go well. But he doesn't want to reduce his treatment now. What do you do ?**

**- You insist, opiate dependent patients have often difficulties to stop psychoactive drugs and treatment.**

**- You accept and respect your patients decision.**

**- You send him back to the tapering protocol , you follow the decrease , and you explane to the patient that he has to follow the objectives predefined.**

- - 1. **Your patient endly accepted to stop buprenorphine. You see him back after the buprenorphine stop. What do you do ?**

**- It is a very risky moment for the patient. You ask him to come back to see you every week.**

**- You ask him to come back to see you in a long time. He has to experience what it feels to live without opiate treatment.**

**- you let him decide. It belongs to him now.**

**We thank you for your involvment in this study. We will send you back the results and a point about literature.**
