# Supplementary material for: French Experience with Buprenorphine : Do Physicians Follow the Guidelines?
Source: PLoS One. 2015 Oct 19;10(10):e0137708. doi: 10.1371/journal.pone.0137708 (PMC4610705; doi:10.1371/journal.pone.0137708)
Supplement: S2 Text — (DOC) [file pone.0137708.s002.doc]

**Cher collègue,**

**Nous menons une étude sur la prescription de Buprénorphine en médecine générale.**

**Beaucoup d'études soulignent l'intérêt de la prescription de buprenorphine en termes de réduction de morbimortalité chez les patients dépendant aux opiacés. Mais les données ne sont pas claires , concernant les indications , la durée du traitement et la manière d'arrêter le traitement. Ce manque de données peut entrainer des difficultés vis à vis de la prescription de BHD.**

**Nos pbjectifs sont :**

**- L'évaluation d'une prescription théorique de BHD sur une étude de cas**

- **L'évaluation de la prescription de BHD par les médecins**
- **La définition d'axes d'amélioration dans la prise en charge de la dépendances aux opiacés en soins primaires**

**Cette étude est menée par le CHRU de Nantes , en articulation avec le réseau addictologie de Nantes et le Département de médecine générale de Nantes.**

**Si vous acceptez de participer, cela vous prendra 5 minutes, les données sont totalement anonymes. A la fin de l'étude, tous les médecins ayant participé seront informés par mail des résultats.**

**Merci beaucoup de votre participation.**

Après cette introduction, les médecins qui acceptaient de participer devait cliquer sur l'item « Répondre au questionnaire » et le questionnaire débutait.

**Quelle âge avez vous?**

**35-45 45-55 - 35 +55**

**Etes vous  :**

**Homme femme**

**Quelle est votre pratique ?**

**Médecine générale Psychiatre Autre : lequel :**

**Avez vous déjà effectué une formation continue en addictologie  ? (universitaire )**

**oui non**

**Avez vous déjà initié un traitement de buprenorphine de vous même ?**

**oui non**

**Combien de patients suivez vous sous traitement par buprenorphine ?**

**1 2-5 +6 aucun**

**Est ce que vous vous sentez à l'aise dans le suivi des patients dépendants aux opiacés ?**

**Oui, absolument, plutot oui plutot non pas du tout non concerné**

**Pensez vous que vous avez suffisamment d'informations concernant le traitement de la dépendance aux opiacés**

**Oui, absolument, plutot oui plutot non pas du tout non concerné**

**Pensez vous que ces informations correspondent avec vos besoins et votre pratique ?**

**Oui, absolument, plutot oui plutot non pas du tout non concerné**

**A propos d'un cas clinique**

**1. Un jeune homme de 27 ans dépendant aux opiacés depuis 3 ans vient vous voir en consultation. Il bénéficie récemment d'un traitement par buprenorphine (subutex ® ) à 12mg/j. Il vous demande combien de temps il devra prendre ce traitement. Que lui répondez vous ?**

- **Quelques mois**
- **environ 3 ans**
- **autre réponse**

1. **Il vient vous voir régulièrement. Vous voulez évaluer son évolution. D'après vous, quelles informations vous semblent importantes pour mesurer l'évolution des conduites addictives ?**

**- Consommation de substances psychoactives ou arrêt**

**- Evaluation multifactorielle incluant l'évaluation sociale, familiale, le statut affectif**

**- La compliance des patients aux soins**

1. **L'amélioration des conduites addictives est longue. Parmi ces variables, lesquelles sont fiables concernant l'évaluation de la capacité des patients à arrêter le traitement de substitution, d'après vous ?**

**- Pas de craving aux opiacés**

**- Pas de consommation opiacés (hormis le traitement de substitution)**

**- Un statut social et professionnel satisfaisant**

1. **Ce patient a déménagé. . Il revient vous voir 5 ans plus tard. Il va bien, il travaille, il a une bonne qualité de vie. Il a encore un traitement de BHD de 4 mg/j. La compliance du traitement est bonne. Il vous demande de renouveler votre prescription. Que lui répondez vous ?**

**- Je pense que vous devriez continuer ce traitement. Il semble vous aider à aller mieux.**

**- Avez vous déjà pensé à la réduction ou l'arrêt du traitement par buprenorphine ?**

**- Actuellement, je ne vois plus d'indications à un traitement par buprénorphine. Je pense que nous pouvons commencer à penser à un arrêt de la buprenorphine.**

- - 1. **Vous avez décidé avec le patient d'arrêter la buprénorphine, quelle est votre attitude ?**

**-Vous discutez et décidez avec le patient des différentes façons de diminuer et arrêter le traitement**

**-Vous arrêtez rapidement le traitement .**

**-Vos définissez un protocole strict avec diminution progressive , avec un objectif d'abstinence opiacés prédéfini.**

- - 1. **Vous avez mené une diminution du traitement. Il a maintenant 2.4mg/day. Le patient vient vous voir, il semble aller bien. Mais il ne souhaite pas réduire le traitement maintenant. Que faites vous ?**

**- Vous insistez, les patients dépendants aux opiacés ont souvent des difficultés à arrêter les substances psychoactives et les traitements.**

**- Vous acceptez et respectez la décision du patient.**

**- Vous le renvoyez au protocole prédéfini. Vous poursuivez la décroissance et vous expliquez au patient qu'il doit suivre les objectifs prédéfinis.**

- - 1. **Votre patient accepte finalement d'arrêter la buprénorphine. Vous le revoyez après l'arrêt de la buprénorphine. Que faites vous ?**

**- C'est une période très à risque. Vous lui demandez de revenir vous voir toutes les semaines.**

**- Vous lui demandez de venir vous voir dans longtemps . Il doit experimenter ce qu'il ressent à vivre sans traitement opiacés.**

**- Vous le laissez décider. Cela lui appartient désormais.**

**Nous vous remercions de votre implication dans cette étude. Nous vous enverrons les résultats par mail et un point sur la littérature.**
