# Supplementary material for: French Experience with Buprenorphine : Do Physicians Follow the Guidelines?
Source: PLoS One. 2015 Oct 19;10(10):e0137708. doi: 10.1371/journal.pone.0137708 (PMC4610705; doi:10.1371/journal.pone.0137708)
Supplement: S3 Text — (DOC) [file pone.0137708.s003.doc]

###
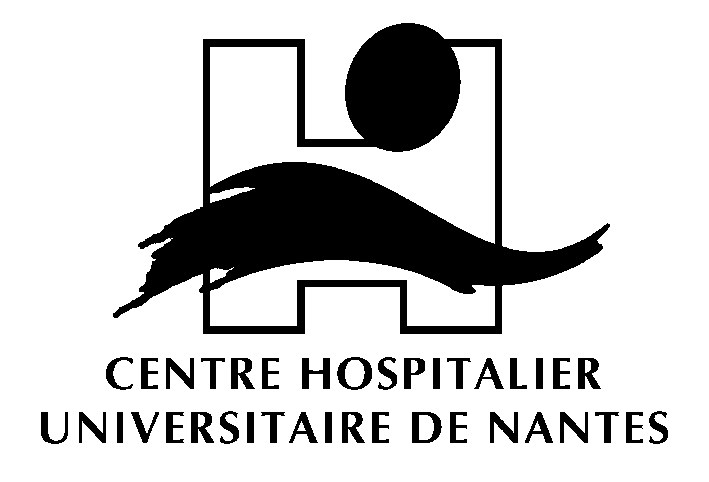
Docteur GUILLOU LANDREAT Morgane

### Addictologie CHRU BREST

**Dr GRALL BRONNEC**

Addictologie CHU NANTES

**Subject :** Clinical research « Survey of the opinions of doctors about prescribing buprenorphine high dosage »

# Contact details :

Anne OMNES


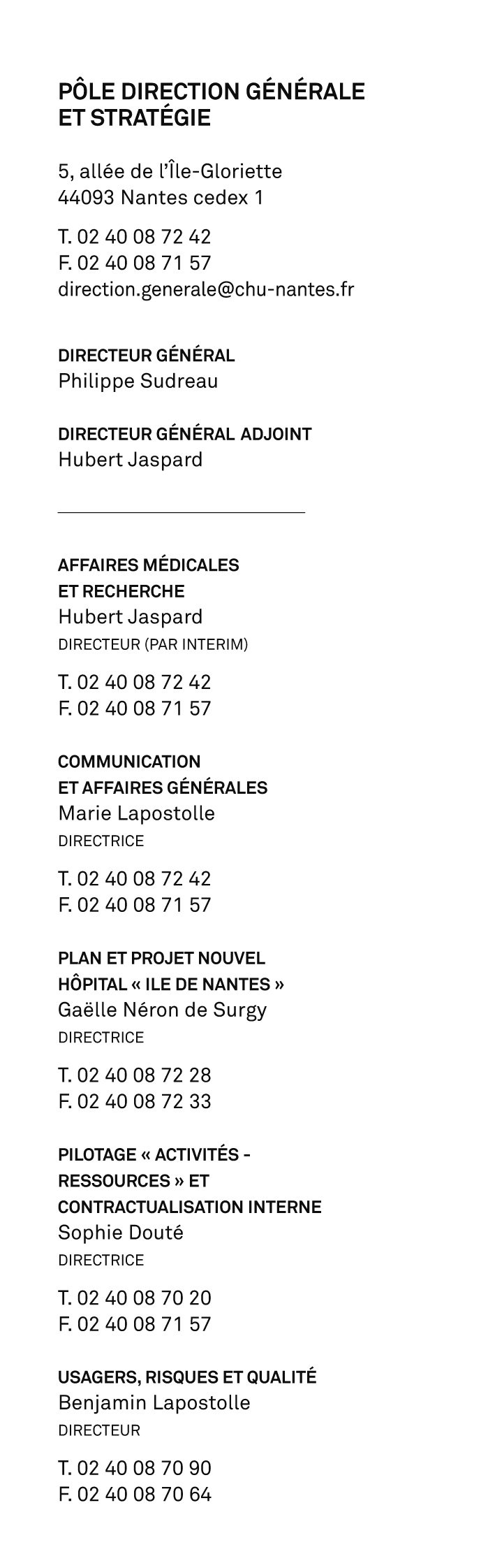
Direction des affaires médicales et de la recherche

Direction de la recherche

Département promotion

**Phone number** +33(0)2.53.48.28.35

**Fax** **number +33(**0)2.53.48.28.36

**To Whom it May Concern**

Hereby, Nantes University Hospital confirms that french legislation about biomedical research did not require competent authorities authorization and ethics committee approval for the clinical research entitled « Survey of the opinions of doctors about prescribing buprenorphine high dosage ». This observational clinical research was carried out in 2010 and concerned data of 193 doctors.

However, according to french legislation, participants were informed about the research and did not oppose to its achievement.

Sincerely yours,

Nantes, March 4^th^, 2015

For the General Director

And by delegation

The Director of medical affairs and research by interim

H. JASPARD
